# Impact of DMEK on visual quality in patients with Fuchs’ endothelial dystrophy

**DOI:** 10.1007/s00417-021-05334-6

**Published:** 2021-09-16

**Authors:** Vanessa Ademmer, Bishr Agha, Mehdi Shajari, Thomas Kohnen, Ingo Schmack

**Affiliations:** 1 Department of Ophthalmology, Goethe-University, Theodor-Stern-Kai 7, 60590 Frankfurt am Main, Germany; 2 Department of Ophthalmology, LMU, Mathildenstraße 8, 80336 Munich, Germany

**Keywords:** DMEK, Fuchs endothelial dystrophy, Visual quality, Scheimpflug imaging

## Abstract

**Purpose:**

To investigate short-term (3 months follow-up) changes in visual quality following Descemet membrane endothelial keratoplasty (DMEK) for Fuchs endothelial dystrophy (FED).

**Methods:**

In this prospective institutional case series, 51 patients that underwent DMEK for FED were included. Assessment included the Quality of Vision (QoV) questionnaire preoperatively, at 1 month, and 3 months after surgery. Secondary outcome measures were anterior segment parameters acquired by Scheimpflug imaging, corrected distance visual acuity (CDVA), and endothelial cell density (ECD).

**Results:**

Glare, hazy vision, blurred vision, and daily fluctuation in vision were the symptoms mostly reported preoperatively. All symptoms demonstrated a significant reduction of item scores for severity, frequency, and bothersome in the course after DMEK (*P* < 0.01). Glare and fluctuation in vision remained to some extent during the follow-up period (median score = 1). Preoperatively, corneal densitometry correlated moderately to weakly with severity of hazy vision (*r*_s_ = 0.39; *P* = 0.03) and frequency (*r*_s_ = 0.26; *P* = 0.02) as well as severity (*r*_s_ = 0.27; *P* = 0.03) of blurry vision. CDVA and central corneal thickness (CCT) did not correlate with visual complains.

**Conclusions:**

Following DMEK for FED, patient-reported visual symptoms assessed by the QoV questionnaire represent a useful tool providing valuable information on the impact of DMEK on visual quality that cannot be directly estimated by morphological parameters and visual acuity only.


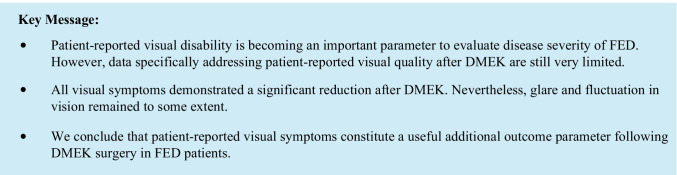


## Introduction

Descemet membrane endothelial keratoplasty (DMEK) has evolved to be the gold standard in the treatment of patients suffering from endothelial dysfunction [1, 2]. Compared to penetrating keratoplasty, DMEK has major advantages, like lower rejection rate, better visual acuity, more predictable refractive outcomes, and lower posterior corneal higher order aberrations [3–5]. In comparison to Descemet’s stripping automated endothelial keratoplasty (DSAEK), which is another commonly applied endothelial keratoplasty technique, DMEK results in better visual acuity and a higher patient satisfaction rate [6, 7]. However, this is currently debated when comparing the visual acuity achieved after ultrathin DSAEK (UT-DSAEK) to DMEK [8].

In patients with FED, pachymetry and visual acuity are mainly used to quantify disease progression as well as functional outcome after DMEK [9, 10]. More recently, corneal light backscatter was introduced as a valuable outcome parameter after DMEK, as reduction in corneal densitometry was associated with improved visual acuity [11]. So far, patient-reported quality of vision was only occasionally included in assessing functional outcome after DMEK. Mainly, it is used for comparing DMEK with other endothelial keratoplasty techniques, like UT-DSAEK [12]. Patient-reported visual disability is becoming an important parameter to evaluate disease severity of FED and led to development of a patient-reported visual disability questionnaire, the Visual Function and Corneal Health Status (V-FUCHS) instrument [13]. In addition, the Quality of Vision (QoV) questionnaire represents a well-established instrument to measure the subjective quality of vision in regard to multiple visual symptoms [14, 15].

Currently, data specifically addressing patient-reported visual quality after DMEK are very limited and do not go into much detail. In order to overcome these limitations and to evaluate an additional functional outcome parameter, we studied the subjective quality of vision over a time period of 3 months in patients undergoing DMEK for FED.

## Methods

### Patients

All patients that underwent DMEK surgery for FED at our department between February 2018 and February 2019 and without any previous corneal surgeries were offered participation in this prospective, observational case series. The study protocol was approved by the institutional review board of the Goethe-University and the tenets of the Declaration of Helsinki were followed throughout the study. Main outcome measures included the Quality of Vision (QoV) questionnaire by McAlinden et al. [14]. Secondary outcome parameters were anterior segment data acquired by Scheimpflug imaging, and clinical information, such as corrected distance visual acuity (CDVA) and endothelial cell density (ECD).

### Quality of Vision questionnaire

Subjective visual quality was assessed before DMEK surgery as well as 1 and 3 months postoperatively. To measure subjective visual quality, all patients answered the QoV questionnaire developed by McAlinden et al. [14]. In this 30-item instrument, 10 symptoms (glare, haloes, starbursts, hazy vision, blurred vision, distortion, double or multiple images, fluctuation in vision, focusing difficulties, difficulty judging distance or depth perception) are each rated in three subscales (frequency, severity, and bothersome). The first seven items of QoV are shown along with pictures demonstrating the respective symptom in the questionnaire. For the three subscales, four response categories with descriptive wording were offered: frequency (never, occasionally, quite often, very often), severity (not at all, mild, moderate, severe), and bothersome (not at all, a little, quite, very). These were transferred into a numeric scale from 0 (never/not at all) to 3 (very often/severe/very) and median values as well as distribution for each answer over time were assessed.

In addition, patients were asked for their overall subjective visual satisfaction on a 5-point scale (1 = very bad, 2 = bad, 3 = moderate, 4 = good, 5 = very good).

### Surgical technique and postoperative treatment

Surgeon-prepared DMEK grafts were used. Graft preparation was performed using a standardized technique as previously described by Melles [2]. Graft diameters of 7.75 and 8.0 mm were selected. For injection of the stained grafts via a glass cartridge (Geuder AG, Heidelberg, Germany), a 2.2-mm corneal incision was used. After unfolding and centration of the DMEK graft over the pupil, sulfur hexafluoride 20% gas was injected into the anterior chamber up to 80 to 90%. A peripheral iridectomy at 6 o’clock was performed in each patient at the beginning of DMEK surgery. After surgery, patients were encouraged to remain in a face-up position for 3–4 days to allow successful graft adherence to the posterior stroma. In situations with partial graft detachment of more than one-third of the graft diameter or more than 3 clock h and clinical signs of corneal edema, rebubbling was performed within 1 to 2 weeks after DMEK using sulfur hexafluoride 20% gas. Control intervals were adjusted, accordingly.

All patients received a standardized postoperative treatment regimen, which consisted of antibiotic eye drops (ofloxacin) 4 times a day for 2 weeks, pilocarpine 1% eye drops applied 3 times a day for the time of anterior chamber gas fill, and topical steroids. For the first 8 weeks, dexamethasone eye drops were applied 6 times a day. Afterwards, these were switched to loteprednol 4 times a day and slowly tapered to a dose of one application per day during the following months.

### Clinical evaluation

Demographic data, visual acuity, and ocular comorbidities were obtained from medical records. In addition, the numbers of patients that required rebubbling and secondary keratoplasty and failed to complete the prospective study were assessed. CDVA was obtained before DMEK as well as 1 and 3 months postoperatively by using decimal charts, which were subsequently converted to logMAR (logarithmic minimal angle of resolution) values. ECD measurements were obtained by specular endothelial microscopic evaluation (Oculus/Nidek CEM-530, Oculus GmbH, Wetzlar, Germany).

### Anterior segment data acquired by Scheimpflug imaging

Scheimpflug tomography (Pentacam AXL, Oculus, Wetzlar, Germany) was used for evaluation of anterior segment parameters. Parameters examined included central corneal thickness (CCT), corneal front and back astigmatism, and average keratometry readings of the anterior (KmF) and posterior surface (KmB) as well as corneal densitometry. Corneal densitometry is a parameter of corneal transparency. Corneal backscattered light is measured and expressed in grayscale units (GSU), ranging from 0 (completely transparent) to 100 (completely opaque). For our study, we assessed the GSU of the total layer (from epithelium to endothelium) for 3 concentric corneal annular zones (0–2 mm zone, 2–6 mm zone, and 6–10 mm zone).

### Statistical analysis

Statistical analysis was performed using Excel for Mac (version 15.37, Microsoft, Inc., Redmond, WA, USA) and SPSS version 25 (IBM Corp., Armonk, NY, USA). The Kolmogorov–Smirnov test was performed for testing normality of data. For normally distributed data, a Student *t*-test for paired values was performed. A two-sided Wilcoxon signed-rank test was used for a non-normal distribution. Correlations were performed using Spearman’s rank correlation coefficient. A *P* value < 0.05 was considered statistically significant.

## Results

A total of 51 patients with a mean age at the time of DMEK surgery of 67.6 ± 11.2 years (range: 35–92 years) were enrolled. Demographic data are summarized in Table 1. Four patients required secondary keratoplasty (repeat DMEK for persistent graft dysfunction, *n* = 3, penetrating keratoplasty for pronounced stromal scarring and insufficient visual recovery, despite a well-functioning DMEK graft, *n* = 1). These patients were excluded from the postoperative analysis. Rebubbling was performed in *n* = 20 patients. A total of 41 patients completed the final follow-up at 3 months after DMEK.

**Table 1:** Demographic data and preoperative clinical parameters

General	
Age (years) mean ± SD (range)	67.6 ± 11.2 (35–92)
Female *n* (%)	30 (59)
Male *n* (%)	21 (41)
Right eye *n* (%)	20 (39)
Left eye *n* (%)	31 (61)
DMEK surgery	
Pseudophakic eyes *n* (%)	22 (43)
Triple DMEK *n* (%)	29 (57)

Preoperative values and postoperative outcomes of CDVA, ECD, and corneal parameters obtained by Scheimpflug tomography are in shown in Table 2.

**Table 2:** Pre- and postoperative values of CDVA, ECD, and corneal parameters obtained by Scheimpflug tomography. Mean ± SD (range)

	Preoperative	1 month	3 months
CDVA^a^ (logMAR)	0.62 ± 0.61 (0.1–3)	0.34 ± 0.46 (0–3)	0.18 ± 0.16 (0–0.7)
ECD (cells/mm^2^)	2775 ± 412 (2280–3600)	2126 ± 482 (580–2951)	1815 ± 529 (504–2781)
CCT (µm)	675.5 ± 138.6 (532–1174)	547.4 ± 48.2 (417–628)	542.8 ± 51.1 (427–660)
CA_ant_ (D)	1.27 ± 0.95 (0.2–3.7)	1.56 ± 1.12 (0.3–5.2)	1.36 ± 0.87 (0.1–4)
KmF (D)	43.5 ± 2.0 (40.1–47)	43.1 ± 1.6 (40.5–46.1)	43.1 ± 1.6 (40.5–46)
CA_post_ (D)	0.63 ± 0.76 (0–2.8)	0.45 ± 0.44 (0.1–2.4)	0.41 ± 0.46 (0–2.5)
KmB (D)	− 5.49 ± 1.04 (− 7.3 to − 2.5)	− 6.4 ± 0.37 (− 7.2 to − 5.8)	− 6.18 ± 0.41 (− 6.9 to − 5.1)
Q post	0.32 ± 0.83 (− 2.94 to 1.56)	0.02 ± 0.34 (− 0.46 to 0.87)	− 0.1 ± 0.34 (− 0.55 to 0.6)
CD (zone)			
0–2 mm	27.1 ± 11.1 (14.2–59.4)	23.3 ± 7.9 (14.8–48.7)	21.4 ± 6.3 (14–43.8)
2–6 mm	22.9 ± 8.9 (14.8–48.4)	21.5 ± 7.1 (13.3–48.8)	19.8 ± 4.6 (13.3–32.7)
6–10 mm	26.7 ± 8.1 (15.4–45.8)	27.9 ± 7.6 (17.7–46.6)	26.7 ± 7.7 (13.7–43.7)

*CDVA*, corrected distance visual acuity; *ECD*, endothelial cell density; *CCT*, central corneal thickness; *CA*_*ant*_, anterior corneal astigmatism; *KmF*, average keratometry readings of the anterior surface; *KmB*, average keratometry readings of the posterior surface; *CA*_*post*_, anterior corneal astigmatism; *Q post*, posterior *Q* value; *CD*, corneal densitometry (total layer). ^a^A total of 4 patients were excluded due to extracorneal visual limitations

### Changes in patient-reported visual quality

Preoperatively, visual symptoms, such as glare, hazy vision, and fluctuation in vision, demonstrated median scores of 2 for frequency (quite often), severity (moderate), and bothersome (quite). For the symptom of blurred vision, a preoperative median score of 2 was observed for severity only (see also Table 3). Three months after DMEK, complains, such as hazy and blurred vision, decreased to median scores of 0 for all items (*P* < 0.001), whereas glare and fluctuating vision decreased significantly at 3 months after DMEK (*P* < 0.001 and < 0.01, respectively), but remained at median scores of 1 for their respective items addressing frequency, severity, and bothersome of these symptoms.

**Table 3:** Course of patient-reported visual symptoms in the QoV questionnaire

	Subscales	Preoperative	Postoperative	
			1 month	3 months
Glare	Frequency	2	1	1
	Severity	2	2	1
	Bothersome	2	1	1
Halos	Frequency	1	0	0
	Severity	1	0	0
	Bothersome	0.5	0	0
Starbursts	Frequency	0.5	1	0
	Severity	0.5	1	0
	Bothersome	0	0.5	0
Hazy vision	Frequency	2	0	0
	Severity	2	0	0
	Bothersome	2	0	0
Blurred vision	Frequency	1	1	0
	Severity	2	1	0
	Bothersome	1	1	0
Distortion	Frequency	0	0	0
	Severity	0	0	0
	Bothersome	0	0	0
Multiple images	Frequency	0	0	0
	Severity	0	0	0
	Bothersome	0	0	0
Fluctuation in vision	Frequency	2	1	1
	Severity	2	1	1
	Bothersome	2	1	1
Focusing difficulties	Frequency	1	0	0
	Severity	1	0	0
	Bothersome	1	0	0
Difficulty judging distance or depth perception	Frequency	1	0	0
	Severity	1	0	0
	Bothersome	1	0	0

Response categories for frequency (0 = never, 1 = occasionally, 2 = quite often, 3 = very often), severity (0 = not at all, 1 = mild, 2 = moderate, 3 = severe), and bothersome (0 = not at all, 1 = a little, 2 = quite, 3 = very). Median scores for each item are shown

Haloes were rated preoperatively with a median frequency and severity of 1 (occasionally and mild, respectively), while, for bothersome, median value was 0.5. Postoperatively, median values for all 3 items addressing haloes decreased significantly to 0 (*P* < 0.01 for severity and frequency, *P* = 0.01 for bothersome at 3 months). Starbursts were noted with a frequency and severity of 0.5 preoperatively, while not being bothersome (median 0). At 1 month post-op, frequency and severity increased to 1 and bothersome to 0.5. At 3 months follow-up, median score was 0 for all three starburst item categories.

For symptoms like focusing difficulties, difficulties judging distance, or difficulties with depth perception (items 25–30), median values of 1 were noted for each item preoperatively. At postoperative follow-up examinations, all decreased to 0 (*P* < 0.01 frequency and bothersome, *P* = 0.02 severity for focusing difficulties; *P* = 0.01 for depth perception at 3 months).

Image distortion and multiple images demonstrated median values of 0 in regard to frequency, severity, and bothersome at all time points.

On the 5-point scale used for rating overall visual quality, median score improved from 2 (bad) to 4 (good) at 1 month after DMEK (*P* < 0.001), which was also the score observed at the final follow-up examination (*P* < 0.001).

### Distribution and course of visual symptoms mostly affected

Preoperatively, scores of 2 or 3 were given by 62% (frequency), 84% (severity), and 77% (bothersome) of patients for glare symptoms. The proportion of scores 1 and 2 increased at the follow-up examinations after DMEK. At 3 months, scores of 2 or 3 were reported by 24% (frequency), 44% (severity), and 24% (bothersome) of participants.

For hazy vision, preoperative symptoms were rated with 3 for frequency, severity, and bothersome by 42%, 24%, and 39%, respectively. At 3 months after DMEK, 3 points were given by no patient for frequency and severity, and very bothersome hazy vision by 10%. The number of patients reporting no hazy vision symptoms (score 0) at 3 months follow-up visit increased to 73% (frequency), 71% (severity), and 73% (bothersome), respectively.

Concerning blurred vision, patients were affected in different ways. Except for the 2-point median score for severity, frequency and bothersome that had median score of 1, all scores (0 to 3) were selected by at least 20% of patients for the item blurred vision, preoperatively. At 3 months after DMEK, there were no complains about blurry vision in 58%, 60%, and 63% of patients in the 3 respective categories frequency, severity, and bothersome (20%, 20%, and 22% preoperatively).

Before DMEK, 58% of patients are affected quite often (score 2) or very often (score 3) by fluctuation of vision, which is severe for 28% and very bothersome for 32%. Three months after DMEK, the respective proportions decreased to 22%, 5%, and 5%.

**Fig. 1:**
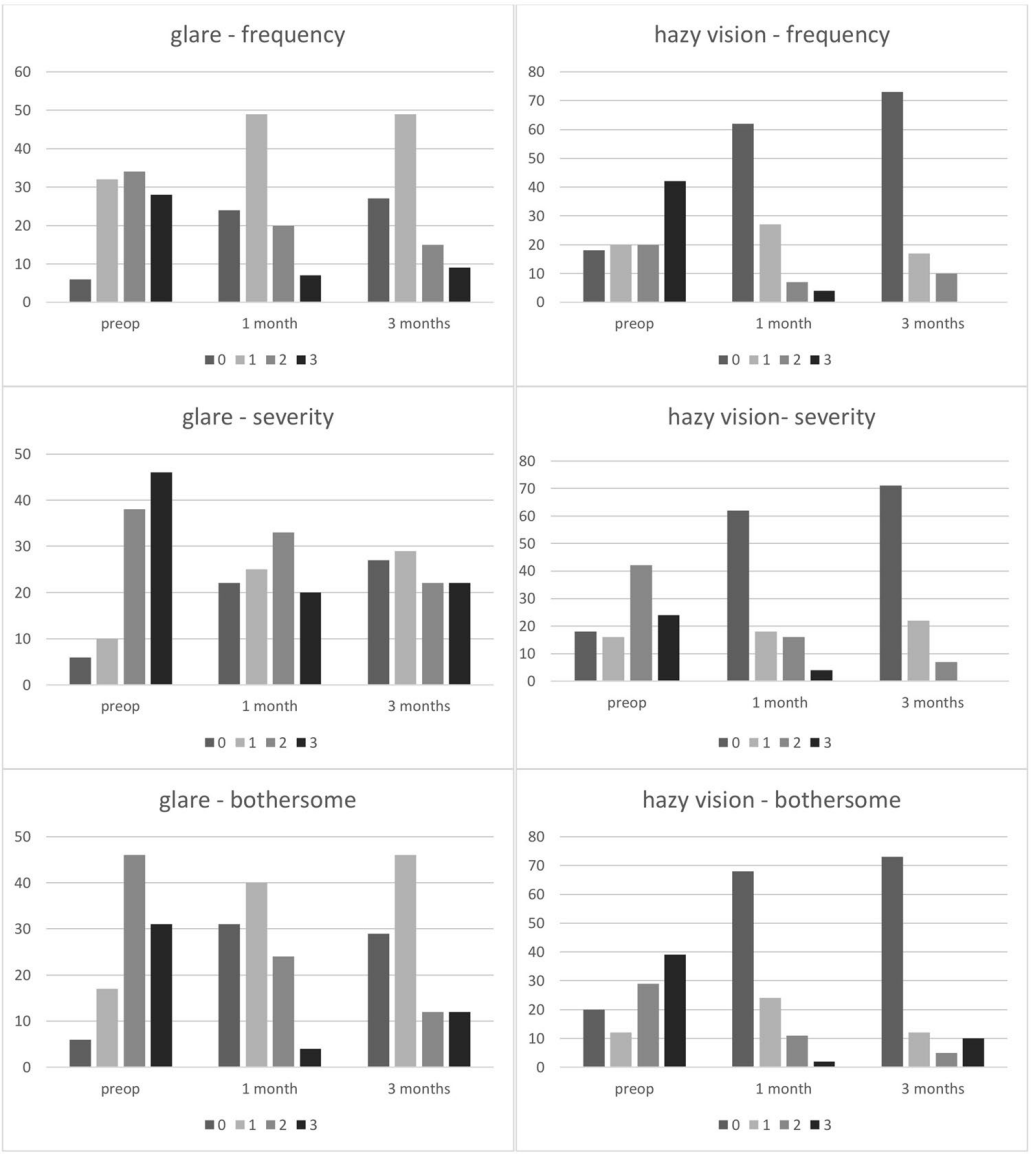
Course of item score distribution of the respective visual symptoms. Distribution of median scores for frequency (0 = never, 1 = occasionally, 2 = quite often, 3 = very often), severity (0 = not at all, 1 = mild, 2 = moderate, 3 = severe), and bothersome (0 = not at all, 1 = a little, 2 = quite, 3 = very). *x*-axis = time-course; *y*-axis = percentage of patients. preop, preoperatively

Distribution and time-course of scores for visual symptoms mostly affected are further summarized in Fig. 1.

### Correlations between visual symptoms and CDVA as well as corneal parameters

Preoperative corneal densitometry values for the central (0–2 mm) zone correlated moderately with the severity of hazy vision (*r*_s_ = 0.39; *P* = 0.03). Additionally, a weak to moderate correlation was found for frequency (*r*_s_ = 0.26; *P* = 0.02) and severity (*r*_s_ = 0.27; *P* = 0.03) of preoperative blurred vision symptoms (Table 4). Postoperatively, only a weak correlation was found between corneal densitometry and glare bothersome (*r*_s_ = 0.17; *P* = 0.03). No significant correlations were found for the 2–6-mm and 6–10-mm zones.

**Table 4:** Spearman’s rank correlation coefficient between corneal densitometry (0–2 mm zone) and visual symptom scores mostly affected

	Corneal densitometry			
			Pre-op	Post-op
Glare	Frequency	*r*_s_	− 0.03	0.01
		*p*	0.89	0.18
	Severity	*r*_s_	0.04	0.01
		*p*	0.43	0.27
	Bothersome	*r*_s_	− 0.03	0.17
		*p*	0.86	0.03
Hazy vision	Frequency	*r*_s_	0.31	0.11
		*p*	0.07	0.07
	Severity	*r*_s_	0.39	0.11
		*p*	0.03	0.26
	Bothersome	*r*_s_	0.28	0.06
		*p*	0.21	0.10
Blurred vision	Frequency	*r*_s_	0.26	− 0.20
		*p*	0.02	0.81
	Severity	*r*_s_	0.27	− 0.29
		*p*	0.03	0.38
	Bothersome	*r*_s_	0.19	− 0.09
		*p*	0.10	0.88
Fluctiation in vision	Frequency	*r*_s_	− 0.14	< 0.01
		*p*	0.16	0.07
	Severity	*r*_s_	− 0.09	< 0.01
		*p*	0.10	0.41
	Bothersome	*r*_s_	− 0.11	0.21
		*p*	0.16	0.09

*r*_*s*_, Spearman’s rank correlation coefficient; *p*, statistically significant; *pre-op*, preoperative; *post-op*, 3 months postoperative

CDVA and CCT did not show significant correlations with subjective visual symptoms.

## Discussion

Although patient-reported visual disability is increasingly often reported as a parameter to evaluate disease severity in FED patients [13] and is relatively well-established for studies concerning intraocular lens implantation [16, 17], comprehensive data on patient-reported visual symptoms and outcome after DMEK are still rare. Therefore, we evaluated the postoperative time-course of patient-reported visual quality in regard to different visual symptoms in 51 patients undergoing DMEK for FED. The QoV questionnaire is a 30-item instrument consisting of 10 different visual symptoms, each rated in three subscales (frequency, severity, and bothersome). It was developed for patients with all types of refractive correction, after laser and intraocular refractive surgery and for eye diseases which cause visual symptoms [14].

Overall, we found that glare, hazy vision, blurred vision, and daily fluctuations in vision were the symptoms mostly affected. Except for blurred vision, which was reported only occasionally and mild in terms of frequency and bothersome, the other symptoms rated as the most disturbing demonstrated relevant impairment of visual quality in each category preoperatively. On the other hand, symptoms like distortion and multiple images seem to be not relevant for FED patients. Haloes and starbursts, visual symptoms that are often assessed in studies evaluating patient satisfaction after intraocular lens implantation [18, 19], were found to play only a minor role in FED patients and to diminish over time in our study. After DMEK, the four most disturbingly rated symptoms (glare, hazy vision, blurred vision, and daily fluctuations in vision) showed a marked improvement, especially hazy and blurred vision. However, for glare and fluctuation in vision, visual disturbance seems to remain at least within 3 months after successful DMEK. As a limitation of our study, it must be stated that these symptoms can also be attributed to the pseudophakic intraocular lens status and ocular surface alterations, like from severe dry eye disease. Additional studies are needed to further investigate the contribution of these extracorneal factors. For the V-FUCHS instrument, a questionnaire developed for assessing visual disability in FED, it is reported that glare and diurnal variation increased with the degree of FED [13]. Another study, which examined morphological parameters, refraction, CDVA, and glare CDVA, concluded that increased morning glare paralleling increased corneal thickness may particularly contribute to visual impairment in FED [20]. This is in accordance with our preoperative findings in the QoV questionnaire for FED patients. Presumably, hazy and blurred vision symptoms additionally observed in our study can be attributed to the severity of corneal decompensation.

Another study evaluating V-FUCHS scores and corneal backscatter in FED patients demonstrated that participants with pronounced posterior corneal backscatter had a high visual disability [21]. In our study, there was a moderate to weak correlation between central corneal densitometry and corneal haziness as well as blurred vision, preoperatively. This finding is consistent with the results of the aforementioned study [21] in regard to FED patients. Interestingly, except for a weak correlation with glare bothersome, we could not find a correlation between corneal densitometry and visual symptoms at the follow-up 3 months after DMEK. Furthermore, there was no correlation between the most affected visual symptoms (glare, hazy vision, blurred vision, and daily fluctuations in vision) and CDVA as well as CCT. Although corneal densitometry is known to correlate with visual acuity after DMEK [22], we could not demonstrate similar correlations between corneal densitometry changes and patient-reported visual symptoms.

The results of our study point out that assessment of visual symptoms can provide valuable additional information that cannot be directly estimated from morphological corneal functional outcome parameters or visual acuity alone. Focusing only on patients undergoing DMEK for FED represents a limitation of our study. Additional studies using larger numbers of patients, extended follow-up times (e.g., up to 2 years), and other forms of endothelial decompensation (e.g., bullous keratopathy) would be of interest to confirm or augment our preliminary results. Although an improvement of visual symptoms was reported by all patients in our study, there might have been also an impact of the rebubbling rate, which was in the upper range compared to the current literature, presumably due to our strict rebubbling criteria [1]. It also might be worthwhile to evaluate additional questionnaires in regard to visual symptoms after DMEK.

In summary, we conclude that patient-reported visual symptoms constitute a useful additional outcome parameter following DMEK surgery in FED patients.

## References

[1] Deng SX, Lee WB, Hammersmith KM (2018). Descemet membrane endothelial keratoplasty: safety and outcomes: a report by the American Academy of Ophthalmology. Ophthalmology.

[2] Melles GR, Ong TS, Ververs B, van der WJ (2006) Descemet membrane endothelial keratoplasty (DMEK). Cornea 25(8):987-990 10.1097/01.ico.0000248385.16896.34 17102683

[3] Maier A-KB, Gundlach E, Gonnermann J (2013). Fellow eye comparison of Descemet membrane endothelial keratoplasty and penetrating keratoplasty. Cornea.

[4] Anshu A, Price MO, Price FW (2012). Risk of corneal transplant rejection significantly reduced with Descemet’s membrane endothelial keratoplasty. Ophthalmology.

[5] Rudolph M, Laaser K, Bachmann BO (2012). Corneal higher-order aberrations after Descemet’s membrane endothelial keratoplasty. Ophthalmology.

[6] Pavlovic I, Shajari M, Herrmann E (2017). Meta-analysis of postoperative outcome parameters comparing Descemet membrane endothelial keratoplasty versus Descemet stripping automated endothelial keratoplasty. Cornea.

[7] Marques RE, Guerra PS, Sousa DC (2019). DMEK versus DSAEK for Fuchs’ endothelial dystrophy: a meta-analysis. Eur J Ophthalmol.

[8] Busin M, Yu AC (2020). The ongoing debate: Descemet membrane endothelial keratoplasty versus ultrathin Descemet stripping automated endothelial keratoplasty. Ophthalmology.

[9] Sun SY, Wacker K, Baratz KH (2019). Determining subclinical edema in Fuchs endothelial corneal dystrophy: revised classification using Scheimpflug tomography for preoperative assessment. Ophthalmology.

[10] Alnawaiseh M, Zumhagen L, Wirths G (2016). Corneal densitometry, central corneal thickness, and corneal central-to-peripheral thickness ratio in patients with Fuchs endothelial dystrophy. Cornea.

[11] Schaub F, Enders P, Bluhm C (2017). Two-year course of corneal densitometry after Descemet membrane endothelial keratoplasty. Am J Ophthalmol.

[12] Ang MJ, Chamberlain W, Lin CC (2019). Effect of unilateral endothelial keratoplasty on vision-related quality-of-life outcomes in the Descemet endothelial thickness comparison trial (DETECT): a secondary analysis of a randomized clinical trial. JAMA Ophthalmol.

[13] Wacker K, Baratz KH, Bourne WM, Patel SV (2018). Patient-reported visual disability in Fuchs’ endothelial corneal dystrophy measured by the Visual Function and Corneal Health Status Instrument. Ophthalmology.

[14] McAlinden C, Pesudovs K, Moore JE (2010). The development of an instrument to measure quality of vision: the Quality of Vision (QoV) questionnaire. Invest Ophthalmol Vis Sci.

[15] Kandel H, Khadka J, Lundström M, Goggin M, Pesudovs K (2017). Questionnaires for measuring refractive surgery outcomes. J Refract Surg.

[16] Kohnen T, Nuijts R, Levy P, Haefliger E, Alfonso JF (2009). Visual function after bilateral implantation of apodized diffractive aspheric multifocal intraocular lenses with a +3.0 D addition. J Cataract Refract Surg.

[17] Kohnen T, Suryakumar R (2020) Measures of visual disturbance in patients receiving extended depth-of-focus or trifocal intraocular lenses. J Cataract Refract Surg 10.1097/j.jcrs.0000000000000364 32818348

[18] Rodov L, Reitblat O, Levy A, Assia EI, Kleinmann G (2019). Visual outcomes and patient satisfaction for trifocal, extended depth of focus and monofocal intraocular lenses. J Refract Surg.

[19] Maxwell A, Holland E, Cibik L (2017). Clinical and patient-reported outcomes of bilateral implantation of a +2.5 diopter multifocal intraocular lens. J Cataract Refract Surg.

[20] Loreck N, Adler W, Siebelmann S (2020). Morning myopic shift and glare in advanced Fuchs endothelial corneal dystrophy. Am J Ophthalmol pii.

[21] Wacker K, Grewing V, Fritz M, Böhringer D, Reinhard T (2020). Morphological and optical determinants of visual disability in Fuchs endothelial corneal dystrophy. Cornea.

[22] Schaub F, Gerber F, Adler W (2019). Corneal densitometry as a predictive diagnostic tool for visual acuity results after Descemet membrane endothelial keratoplasty. Am J Ophthalmol.

